# Online Health Information Seeking Behaviors Among Older Adults: Systematic Scoping Review

**DOI:** 10.2196/34790

**Published:** 2022-02-16

**Authors:** Yuxiang Chris Zhao, Mengyuan Zhao, Shijie Song

**Affiliations:** 1 School of Economics and Management Nanjing University of Science and Technology Nanjing China; 2 Business School Hohai University Nanjing China

**Keywords:** older adults, online health information seeking, health information behavior, aging technology, systematic scoping review

## Abstract

**Background:**

With the world’s population aging, more health-conscious older adults are seeking health information to make better-informed health decisions. The rapid growth of the internet has empowered older adults to access web-based health information sources. However, research explicitly exploring older adults’ online health information seeking (OHIS) behavior is still underway.

**Objective:**

This systematic scoping review aims to understand older adults’ OHIS and answer four research questions: (1) What types of health information do older adults seek and where do they seek health information on the internet? (2) What are the factors that influence older adults’ OHIS? (3) What are the barriers to older adults’ OHIS? (4) How can we intervene and support older adults’ OHIS?

**Methods:**

A comprehensive literature search was performed in November 2020, involving the following academic databases: Web of Science; Cochrane Library database; PubMed; MEDLINE; CINAHL Plus; APA PsycINFO; Library and Information Science Source; Library, Information Science and Technology Abstracts; Psychology and Behavioral Sciences Collection; Communication & Mass Media Complete; ABI/INFORM; and ACM Digital Library. The initial search identified 8047 publications through database search strategies. After the removal of duplicates, a data set consisting of 5949 publications was obtained for screening. Among these, 75 articles met the inclusion criteria. Qualitative content analysis was performed to identify themes related to the research questions.

**Results:**

The results suggest that older adults seek 10 types of health information from 6 types of internet-based information sources and that 2 main categories of influencing factors, individual-related and source-related, impact older adults’ OHIS. Moreover, the results reveal that in their OHIS, older adults confront 3 types of barriers, namely individual, social, and those related to information and communication technologies. Some intervention programs based on educational training workshops have been created to intervene and support older adults’ OHIS.

**Conclusions:**

Although OHIS has become increasingly common among older adults, the review reveals that older adults’ OHIS behavior is not adequately investigated. The findings suggest that more studies are needed to understand older adults’ OHIS behaviors and better support their medical and health decisions in OHIS. Based on the results, the review proposes multiple objectives for future studies, including (1) more investigations on the OHIS behavior of older adults above 85 years; (2) conducting more longitudinal, action research, and mixed methods studies; (3) elaboration of the mobile context and cross-platform scenario of older adults’ OHIS; (4) facilitating older adults’ OHIS by explicating technology affordance; and (5) promoting and measuring the performance of OHIS interventions for older adults.

## Introduction

During the past decade, the rapid development of information and communication technologies (ICTs) has increased laypeople’s access to health information sources and is constantly reshaping their health information–seeking behaviors [1]. Online health information seeking (OHIS) serves multiple purposes, such as understanding disease symptoms, assessing disease risks, finding treatment choices, managing chronic conditions, and preparing for patient-doctor communication [2]. Studies have revealed that OHIS has become one of the most common everyday life experiences across the entire lifespan [3].

In recent decades, the aging of the world population has led to significant demographic transitions that have never occurred before in human history. Societies with large aging populations face great challenges to their health care sectors with respect to an increasing prevalence of chronic conditions among older adults and a sharply rising demand for health care resources. As older adults are more likely to experience illness and chronic conditions than younger people, they have a greater need for health information [4]. With the world population aging, increasing numbers of health-conscious older adults are seeking health information to make better-informed health decisions [5]. Many hopes are placed on ICTs to empower the aging population, promote public health, and alleviate the burden of health care systems. However, there is some skepticism regarding whether older adults really benefit from current technological advancements [6]. Although some studies have found that the adoption and use of ICTs to address health concerns have remained at a relatively low rate among older adults [7], other studies suggest that older adults are increasingly engaged in internet surfing [8]. These mixed results suggest that the OHIS behavior of older adults is still insufficiently investigated.

Despite scattered empirical studies on the topic, few scoping or systematic reviews have directly addressed the OHIS behaviors of older adults and synthesized this body of knowledge. Chang and Huang [9] recently reviewed antecedents that predict general consumers’ OHIS behaviors (ie, health status, self-efficacy, health literacy, availability, credibility, emotional responses, and subject norms). Although the review found that age is a significant moderator of the correlations between the antecedents and OHIS, it provided few details on older adults’ health information behaviors. Hunsaker and Hargittai [8] synthesized quantitative literature on general internet use among older adults. Although their review addressed the relationship between older adults’ health and internet use, OHIS was neither specified nor teased out from the general internet use behaviors. Therefore, the type of health information sought by the participating older adults and the factors that influenced older adults’ OHIS reported in the literature are unclear. Waterworth and Honey [10] reviewed 8 empirical studies of OHIS among older adults and discussed facilitators of and barriers to older adults’ OHIS. However, the number of studies included in this review was limited, and it can hardly provide a comprehensive understanding of OHIS among older adults.

Gaps in the existing research indicate that a systematic scoping review on older adults’ OHIS is necessary because it will not only enhance our knowledge of human information behaviors and practices but will also inform better health information system designs and ensure better information services for older adults. Motivated by the existing research gaps, this systematic scoping review examines the state of research on older adults’ OHIS and reveals the types and sources of health information that the older adults seek, factors that influence older adults’ OHIS, barriers to older adults’ OHIS, and interventions that are available. The purpose of this systematic scoping review is to provide our readers with an overview of how OHIS among older adults has been studied and present implications for future research. It aims to answer the following questions:

1. What types of health information do older adults seek and where do they seek health information on the internet?

2. What are the factors that influence older adults’ OHIS?

3. What are the barriers to older adults’ OHIS?

4. How can we intervene and support older adults’ OHIS?

## Methods

### Literature Search

This review follows the guidelines of the PRISMA-ScR (Preferred Reporting Items for Systematic Reviews and Meta-Analyses Extension for Scoping Reviews) [11]. We were also inspired by the recommended framework for conducting systematic reviews in information-related fields by Okoli [12]. The bibliographic database search strategies were developed after consulting an academic librarian at the first author’s university.

First, we searched the following databases: Web of Science; Cochrane Library database; PubMed; MEDLINE; CINAHL Plus; APA PsycINFO; Library and Information Science Source; Library, Information Science and Technology Abstracts; Psychology and Behavioral Sciences Collection; Communication & Mass Media Complete; ABI/INFORM; and ACM Digital Library. These databases were chosen because they cover the academic disciplines (eg, medicine, medical informatics, communication, psychology, and information and library science) that are most likely to study older adults’ OHIS behaviors. Second, the search queries contained the following categories and keywords: people (older adults, elderly, aging, senior, seniors, older people, aged 60, aged 65), behavior (find, search, seek, access, retrieve), place (internet, online, web), object (information), and attribute (health, medicine, drug, nutrition, diet, wellness, illness). Specific queries were run in the topic, title, and abstract fields, depending on the database (see Multimedia Appendix 1). The initial search was performed in November 2020. Third, we captured additional articles using Google Scholar by tracking the citations and references in the articles found in the databases and in other relevant reviews. In addition, we supplemented relevant articles by searching Google Scholar directly. All the studies identified during the database searches were imported into the reference management software Zotero, and duplicates were removed.

### Eligibility Criteria

We developed a series of inclusion and exclusion criteria to identify articles relating to older adults’ OHIS behaviors. The inclusion criteria were as follows: (1) The articles should pertain to health-related contexts, including areas such as health, mental health, diet, and nutrition. (2) The article should describe OHIS behaviors (eg, general OHIS, selection and use of health information sources, and adoption and use of health information). (3) The article should focus on older adults (Note that although the search strategies indicated 2 commonly accepted lower age boundaries, 60 and 65 years, to identify older adults, it did not exclude other ways to describe the population); studies that clearly mentioned the population of older adults or contained explicit, equivalent claims were eligible. (4) The research should be empirically based. (5) The articles should have been published in a peer-reviewed journal or in conference proceedings. (6) When we identified more than 1 paper published by the same author on the same topic, we selected only the most recent one. (7) The articles should be written in English.

Our exclusion criteria were as follows: (1) The articles did not pertain to a health-related context. (2) The articles were not about OHIS behaviors; for instance, some articles focused only on general ICT use or adoption behaviors, were more concerned with technology-related rather than information-related issues or addressed only older adults’ health literacy or eHealth literacy and did not investigate their OHIS. (3) The articles did not focus on older adults; we specifically excluded articles that treated age merely as a predictor or moderator in studying the OHIS of the general population, as it is evident that age influences people’s OHIS behaviors. (4) The articles were not based on empirical research; this criterion helped eliminate opinion pieces, brief communications, editorial commentaries, and reviews. (5) The articles were not peer-reviewed (eg, a self-archived manuscript). (6) The articles were not written as full papers (eg, abstracts, posters, or letters). (7) The articles were not written in English.

### Screening Procedure

The procedure for screening articles was based on the eligibility criteria. The initial search used database search strategies and identified 8047 publications. After duplicates were removed, the data set consisted of 5949 publications for screening.

The screening involved 3 stages. In the first stage, all the 3 authors reviewed the titles and abstracts of a sample of 300 articles from the search results, and then discussed and refined the screening criteria. In the second stage, we selected another 300 articles randomly from the search results as a test set. The feasibility criteria were verified independently by 2 of the authors (SS and MZ). Intercoder agreement (κ=0.816) indicated satisfactory reliability. Discrepancies were discussed and resolved by involving the third author (YZ), and the eligibility criteria were further refined accordingly. In the third stage, author MZ screened the remaining articles based on the eligibility criteria using the titles and abstracts, and author SS validated the results. Discrepancies were resolved by involving author YZ. The whole screening procedure resulted in 279 articles for full-text analysis.

To read and code the full-length articles downloaded from the databases, we used the MAXQDA 2020 software, which is designed for analyzing computer-assisted qualitative and mixed methods data, texts, and multimedia data. During the full-text analysis, we excluded 211 articles by applying the eligibility criteria. The remaining 68 articles were retained, and 8 more eligible articles were identified through citation tracking with the assistance of Google Scholar. In total, 75 articles were selected for the systematic scoping review.

### Data Extraction and Analysis

We used Excel (Microsoft Corporation) to extract and record the basic information of the articles in the sample, including the author(s), title, publication year, publication name, and publication type (eg, journal vs conference). We used thematic content analysis in an iterative manner to identify the evidence regarding our research questions [13]. Several lists of codes were generated during 2 rounds of full-text coding procedures. In the first round, all the authors participated in the open and selective coding processes until a coding schema emerged and converged. In the second round, MZ coded the full texts by applying the coding schema, and SS validated all the codes. The intercoder reliability of the thematic content analysis reached 85%. Discrepancies were solved by involving YZ in the discussion.

## Results

### Basic Characteristics of the Included Articles

**Figure 1:**
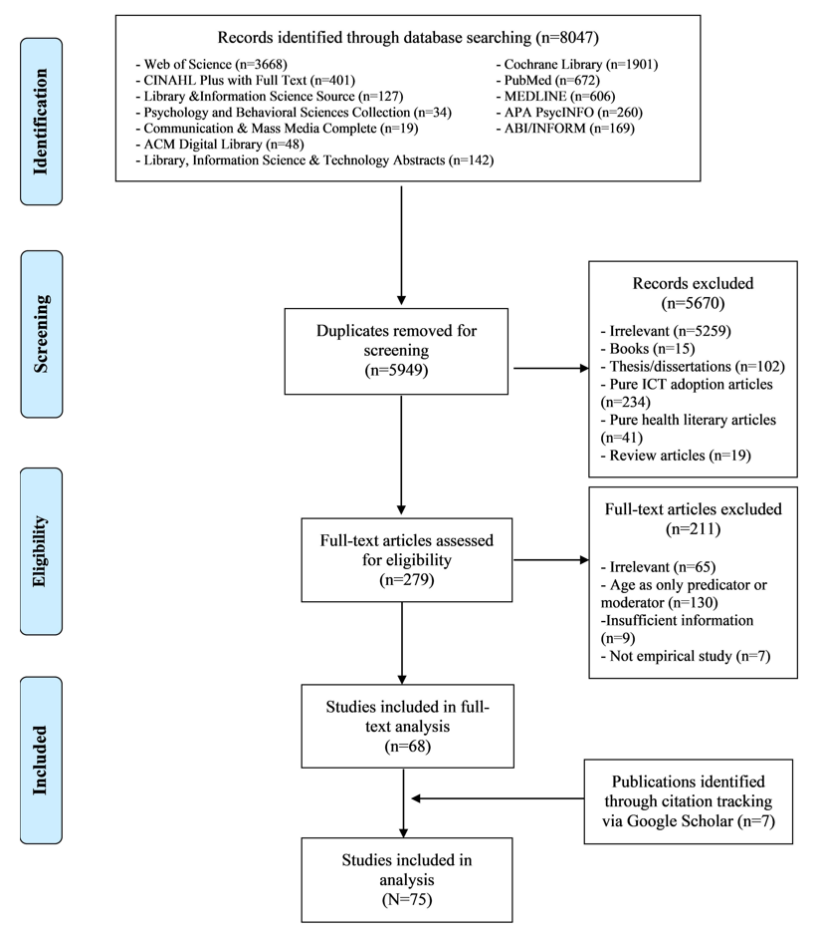
Screening procedure. ACM: Association for Computing Machinery; APA: American Psychological Association; CINAHL: Cumulative Index to Nursing and Allied Health Literature; ICT: information and communication technology.

**Figure 2:**
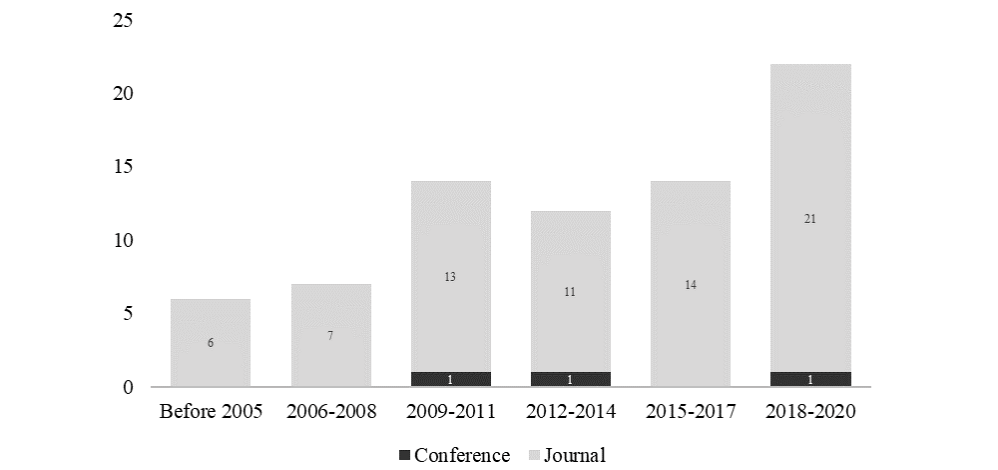
Distribution of publication years.

After screening, the final sample consisting of 75 articles was obtained, as shown in Figure 1. The articles were published between 1997 and 2020 (see Multimedia Appendix 2). Trend observations revealed that the number of publications in this subject area increased over time and that the OHIS of older adults began to receive considerable attention in the last 3 years (see Figure 2). The articles in the sample were mostly published after 2006 (n=69, 92%), which relates closely to the boom in social media. Of all the articles, 72 (96%) were published in journals, and the remaining 3 (4%) were published in conference proceedings. The articles originated from 17 countries (based on the first author’s affiliations), with the top 3 being the United States (n=44, 58.67%), Australia (n=5, 6.67%), and China (n=4, 5.33%). The top 4 journals publishing these articles include the Journal of Medical Internet Research (n=8, 10.67%), Educational Gerontology (n=4, 5.33%), Journal of Health Communication (n=3, 4%), and Library & Information Science Research (n=3, 4%), indicating the multidisciplinary nature of the sample.

The systematic scoping review first investigated how the included 75 articles defined the target population of older adults. The cutoff ages for defining older adults were determined. More than half of the articles used samples of older adults aged above 60 years. Furthermore, 16 articles (21.33%) defined older adults as those aged 65 years and above, and 23 (30.67%) had cutoff ages ranging from 60 to 64 years. In addition, we noted some papers that defined the older adult group more loosely. For example, the cutoff age in 17 articles (22.67%) ranged from 50 to 54 years, and 14 articles (18.67%) used samples with minimum ages ranging from 55 to 59 years. Moreover, 5 of the articles (6.67%) did not specify precise age distributions.

The research methods varied across the 75 studies. Regarding methodological approaches, we found that 45 studies (60%) used quantitative approaches, 22 (29.33%) employed qualitative approaches, and 8 (10.67%) were based on mixed methods designs, using a combination of quantitative and qualitative methods. As for specific methods, surveys (n=28, 37.33%) and interviews or focus groups (n=25, 33.33%) were the primary methods used, followed by secondary data analysis (n=6, 8%) and experiments (n=4, 5.33%). In terms of data sources, most of the studies were based on primary data (n=65, 86.67%) and a few on secondary data (n=10, 13.33 %). Concerning the types of data, we found 59 studies (78.67%) based on cross-sectional data and 16 (21.33%) based on longitudinal data.

### Internet-Based Health Information Types and Sources

Information types and information sources are 2 frequently reported aspects of information in OHIS studies [14]. For our analysis, we adapted the typologies of health information types from Kent et al [15] and Ramsey et al [16]. The results presented in Table 1 suggest that older adults often search the internet for information on specific diseases because they want to obtain a general idea of their condition before diagnosis or treatment so that they know what to expect and can be better prepared to face stressful situations [17]. The health problems mentioned in these 75 articles are mainly cancer (n=10, 13.33%), mental health problems (n=5, 6.67%), chronic conditions (n=4, 5.33%), and physical diseases (n=4, 5.33%). Aside from this disease information, the most frequently mentioned types of information are related to medication or treatment, nutrition or exercise, medical research, disease symptoms, and health promotion. Some articles mentioned that older adults also use the internet to seek support groups or interpersonal advice, health insurance information, health news, and health policy information. Of note is that more than half of the articles (n=40, 53.33%) used the umbrella term health information, without specifying any type of health information content. Furthermore, the types of content were not mutually exclusive. For example, a single article might mention more than 1 type of information (eg, older adults seeking information for cancer-related symptoms and medication).

**Table 1:** Types of health information mentioned in the articles (N=75).

Type of health information	Number of articles (n)
General health information	40
Specific diseases	23
Medication/treatment	21
Nutrition/exercise	13
Medical resource	12
Disease symptoms	9
Health promotion	8
Support groups/interpersonal advice	4
Health insurance	4
Health news/policies	3

Most of the articles in the sample (n=58, 77.33%) used the general internet to represent all the web-based sources of health information. Further, 26 articles (34.67%) described health websites as sources of internet-based health information for older adults; among these, the owners of the websites varied, consisting of educational, commercial, government, and nonprofit entities. Moreover, general search engines such as Google were the third most frequently mentioned sources in the studies (n=17, 22.67%), suggesting that older adults often use general search engines to start OHIS [18-20]. Further, 11 articles (14.67%) mentioned older adults’ use of social media (eg, Facebook, Twitter) and blogs in OHIS. Only 3 articles (4%) addressed older adults’ use of patient portals, and 2 articles (2.67%) were about older adults’ use of mobile internet services. Table 2 shows the health information sources mentioned in the studies.

**Table 2:** Internet-based health information sources mentioned in the studies (N=75).

Source of internet-based health information	Number of articles (n)
General internet	58
Health websites (eg, WebMD, Mayo Clinic)	26
General search engines (eg, Google, Yahoo)	17
Social media/blogs (eg, Facebook, Twitter)	11
Patient portals	3
General mobile	2

### Factors That Influence Older Adults’ OHIS Behaviors

Among the 75 articles, 35 (46.67%) treated OHIS as a variable or construct. These articles quantitatively measured OHIS with various scales or proxy variables. Among them, 27 (36%) regarded OHIS as a dependent variable and explored the antecedents of older adults’ OHIS. Further, 4 (5.33%) treated OHIS as an independent variable, and the remaining 4 (5.33%) treated OHIS as neither a dependent nor an independent variable but provided only descriptive analyses. Because the articles that employed quantitative approaches primarily concerned the antecedents of older adults’ OHIS, we summarize the antecedents in Table 3.

We summarize the main influencing factors that appeared in the investigations. The antecedents of older adults’ OHIS fall mainly into 2 categories, namely individual-related characteristics and source-related characteristics. Within the individual-related characteristics, 12 subcategories were observed, including demographics, anxiety, beliefs, attitudes, self-efficacy, personality, health status, medical history, health care service availability, source experience, health literacy and motivations. Among the source-related characteristics, credibility, usefulness, and ease of use were the 3 most frequently mentioned factors.

**Table 3:** Factors influencing older adults’ online health information seeking behaviors.

Influencing Factors					Studies
**Individual-related characteristics**					
	**Demographics**				
		Socioeconomic status	[18,21-35]		
		Education	[18,21-41]		
		Gender	[18,21-27,29-34,36-43]		
		Marriage	[21,22,24,29,31,34,36,37,42,43]		
		Race/ethnicity	[23,29,30,33-35,37,38,40,44]		
		Place of residence	[24,30,36,41]		
		No. of children	[36]		
		Living with children	[33,35]		
	**Anxiety**				
		ICT^a^-related anxiety	[18,42,45]		
		Disease-related fears	[31,46]		
		Perceived susceptibility	[21]		
	**Beliefs**				
		External control	[45]		
		Internal locus of control	[28]		
		Fatalistic belief	[31,40]		
	**Attitudes**				
		Attitudes on patient-doctor relationship	[18]		
		Reliance on and compliance with doctor’s decisions	[18]		
		Attitudes on ICT use	[39,42]		
		Attitudes on internet-based health information	[38,45]		
		Attitudes on patient-doctor relationship	[18]		
	**Self-efficacy**				
		Self-efficacy in health	[36]		
		Self-efficacy in learning	[24,42]		
		Self-efﬁcacy in ICT use	[42,45]		
	**Personality**				
		Big five	[36,42]		
		General values and life goals	[41]		
	**Health status**				
		General health conditions	[18,21,28-30,32,38,41,42]		
		Physical health	[22,33-39]		
		Mental health	[31,34,36,37]		
		Chronic conditions	[22,30-36,43,44]		
	**Medical history**				
		Personal medical history	[21]		
		Family medical history	[21,31]		
	**Health care service availability**				
		Health care use	[34,36]		
		Health insurance status	[34]		
		Medical financial burden	[33]		
	**Source experience**				
		Experience in internet use	[18,38,39,45]		
		Internet use frequency	[26,34,39]		
		Experience with online health information seeking	[29]		
		Experience in ICT use	[35,42]		
		Internet knowledge	[27]		
	**Health literacy**				
		Health literacy	[24,33,43]		
		eHealth literacy	[27,29]		
	**Motivations**				
		Health information needs	[18]		
		Health information orientation	[27]		
		Health information overload	[46]		
		Subjective norms	[39,45]		
**Source-related characteristics**					
	**Credibility**				
		Trustworthiness	[28,38]		
		Relevance	[45]		
		Output quality	[45]		
		Result demonstrability	[45]		
	**Usefulness**				
		Perceived usefulness of internet health information	[45]		
		Perceived usefulness of internet use	[28,39]		
		Perceived importance of health information	[28]		
	**Ease of use**				
		Perceived ease of use of internet health information	[45]		
		Perceived ease of internet use	[39]		
		Computer playfulness	[45]		
		Perceived enjoyment	[45]		

^a^ICT: information and communication technology.

### Barriers to OHIS of Older Adults

Rather than treating OHIS as a variable, 40 of the 75 articles (53.33%) treated OHIS as a process. Of these studies, 29 (38.67%) explored the barriers that older adults encounter during OHIS. The results suggest that older adults may experience many barriers preventing successful OHIS, as shown in Table 4. In the prior studies, we identified 3 main types of barriers (ie, individual, social, and ICT), 11 subtypes, and 38 specific issues.

**Table 4:** Barriers to older adults’ online health information seeking behavior.

Barrier types				Studies
**Individual barriers**				
	**Functional decline**			
		Vision impairment	[20,34]	
		Physical challenges (eg, back pain, knee injury)	[47,48]	
		Illness conditions	[32,35,36]	
	**Low literacy**			
		English language literacy	[49,50]	
		Basic health knowledge	[51,52]	
		Digital literacy	[53,54]	
		Information literacy	[52,55,56]	
		Health literacy	[24,33,43]	
		eHealth literacy	[27,57]	
	**Low self-efficacy**			
		Low efficacy and anxiety associated with computer use	[18,49,58,59]	
		Low efficacy in reading and learning	[49,60,61]	
		Low efficacy in OHIS^a^	[62,63]	
		Low efficacy in health information evaluation	[55,62]	
	**Negative attitudes**			
		Attitude toward internet use	[39]	
		Attitude toward technology	[42]	
		Privacy concerns	[20,61,64]	
	**Health beliefs**			
		External locus of control	[45]	
		Fatalistic beliefs	[31]	
**Social barriers**				
	**Social stigmas**			
		Stigma of mental health problems	[65]	
		Stigma of sex-related health problems	[66]	
	**Lack of social support**			
		Lack of informational support	[66,67]	
		Lack of organizational support (eg, health care services)	[17,50,68]	
		Lack of instrumental support (eg, instructions on computer use)	[57,65]	
		Lack of intergenerational support (eg, not living with children)	[49,69]	
		Lack of peer support (eg, hard to get support from friends)	[70,71]	
**ICT^b^ barriers**				
	**Lack of IT^c^ infrastructure**			
		Lack of ICT devices	[29]	
		Low accessibility to medical records	[71]	
	**Problematic information quality**			
		Misinformation	[64,72]	
		Conflicting health information	[73,74]	
		Irrelevant information	[65,73]	
	**Information overload**			
		Overwhelming health information on the internet	[20,48,71]	
		Overwhelming extraneous information and pop-ups	[58,64,70]	
	**Unsatisfactory user experiences**			
		Unsatisfactory interactivity and navigability	[75,76]	
		Unsuitable font sizes	[72,75]	
		Dense text and lack of visual elements	[76,77]	
		Confusing layouts	[51,72,75]	
		Insufficient ease of use	[39,45,78]	
		Frustrating user experiences	[51,56,59]	

^a^OHIS: online health information seeking.

^b^ICT: information and communication technology.

^c^IT: information technology.

Regarding individual barriers, some studies found that older adults’ OHIS could be hindered by age-related functional decline, including vision impairment, poor eye-hand coordination, physical challenges (eg, back pain), and illness. Moreover, some studies reported several aspects indicating low literacy among older adults that prevented effective OHIS, including limited English language skills, lack of basic health knowledge, limited digital literacy, undeveloped information literacy, and low health or eHealth literacy. Moreover, some studies found that older adults’ perceptions of low self-efficacy regarding computer use, reading, learning, and evaluation of health information reduced their willingness toward OHIS. Other findings revealed that negative attitudes toward internet use or general technology and privacy concerns about using technology decreased older adults’ intentions to search information on the internet. The results also revealed that beliefs regarding the external locus of the control of health care and fatalistic beliefs reduced older adults’ active OHIS.

As for social barriers, studies suggested that older adults may have some social stigma concerning OHIS when it comes to mental and sex-related health problems. Moreover, older adults often report a lack of social support in their OHIS, including informational, organizational (eg, health care services), instrumental (eg, instructions on computer use), intergenerational (eg, support from children), and peer support (eg, support from friends).

In terms of ICT use, analysis of the studies revealed that many older adults do not possess information technology devices, and they reported low accessibility to medical records. Moreover, the quality of general health information on the internet is problematic. Older adults are likely to encounter misinformation, conflicting information, and irrelevant information during their OHIS. Furthermore, they often confront information overload when reading health information due to overwhelming amounts of irrelevant information or pop-ups. Moreover, older adults’ OHIS may lead to some unpleasant and frustrating user experiences, such as unsatisfactory interactivity and navigability, unsuitable font sizes, dense text lacking visual elements, confusing layouts, and complicated site designs.

### Interventions for Older Adults’ OHIS

Given the abovementioned barriers, it is essential to provide older adults with additional support to facilitate their OHIS. We identified 11 studies (14.67%) among the 75 that used educational training programs to facilitate and intervene in older adults’ OHIS, as shown in Table 5. Among these, 10 of the 11 studies provided offline workshops, and 1 conducted an online workshop. The offline workshops were conducted in community settings (eg, public libraries, schools, or medical centers) and included face-to-face instruction. We identified only 1 study that used an internet-based tutorial to improve older adults’ ability to distinguish high-quality internet-based health forums from low-quality ones. Among the 11 articles, 9 described training programs with multiple sessions, each lasting 2 to 3 hours, and the duration of the programs varied from 1 to 4 months; the other 2 studies used 1-time training sessions.

**Table 5:** Interventions to support older adults’ online health information seeking behaviors.

Study	Main objective	Intervention format	Intervention setting	Intervention evaluation measures
Malone et al [20]	To improve the health literacy skills of older adults	Educational program: Participants could attend every class offered at their library or could select the classes most appropriate to their personal needs and interests. No. of participants: 110	5 local libraries	Method: Pre- vs postsession surveys Qualitative analysis with descriptive statistics: Participants’ confidence in their OHIS^a^ increased, and the overall response to the program was positive.
Bertera et al [67]	To increase access to and use of 2 prominent health websites: MedlinePlus.gov and NIHSeniorHealth.gov	2-step training: (1) Training of internet navigators: 13 hours of basic training in computer skills over 13 weeks, plus a 4-hour specific training on 2 health websites and training on how to support peers during the process. No. of participants: 8 (2) Training of older adults living in affordable housing: 2-hour session on basic computer skills and use of 2 specific health websites. No. of participants: 42	A computer learning center located in the community	Method: Pre- vs posttest surveys, face-to-face interviews A significant improvement in the ability to use a computer or navigate the web was observed (*P*<.001). The average navigational skills self-efficacy score for health web sites (*P*<.001) and computers (*P*<.001) improved.
Chu et al [68]	To assist older adults with retrieving and evaluating health information resources on the internet	Educational program: 2-hour sessions once a week over 5 weeks. Partnering with Seniors for Better Health: Classes included 2 components, computer literacy and health information search strategies. No. of participants: 112	A computer lab offered at a facility of the YWCA^b^ in Houston	Method: Pre- vs posttest surveys; survey conducted 6 weeks after training Participants experienced reduced computer anxiety and increased confidence and sense of self-efficacy when retrieving and evaluating internet-based health information (*P*<.001).
Campbell [79]	To improve the ability to locate health information	Workshops: 2-hour sessions once a week over 5 weeks The sessions used constructivist teaching techniques and self-directed learning. No. of participants: 70	A large suburban public library and 2 community centers for older adults	Method: Posttest interview Qualitative assessment by asking participants questions such as “Did your levels of participation in your health care change since you began using the internet?”
Campbell and Nolfi [80]	To teach older adults to access health care information on the internet	Workshops: 2-hour sessions once a week over 5 weeks No. of participants: 42 Follow-up survey 1 year after the workshops No. of participants: 27	A large suburban public library and 2 community centers for older adults	Method: Pre- vs. posttest surveys; survey 1 year after the training Statistically significant differences were found between baseline and 5-week follow-up results for MHLC^c^ in males (*P*=.02) and females (*P*=.05), as well as for Krantz HOS^d^ information seeking scores (*P*=.05).
Hoffman-Goetz et al [81]	To improve the internet search skills of adults aged 50 years and older	Workshops: 2-hour workshops once a month, over 4 months. The maximum number of participants per workshop was 15. Total No. of participants: 44	Public library with computer stations, led by a researcher, librarian, and university-based investigators	Method: Pre- vs posttest surveys Participants’ search difficulty decreased after the workshops (*P*<.001). Participants’ understanding of the internet improved after the workshops (*P*<.001).
Leung et al [82]	To improve basic skills for searching health information on the internet	Workshops: 3-hour training course The number of participants per workshop was 30. Total No. of participants: 88	Local university and company, instructed by nursing lecturer and students	Method: Postsession telephone interviews 1 month after the workshop Participants’ confidence level in seeking health information was significantly associated with the level of satisfaction with the workshop (*P*<.001).
Campbell [83]	To improve health literacy skills among low-income, minority, and older adults	Workshops: 2-hour sessions once a week over 5 weeks No. of participants: 36	Computer labs in 2 low-income, minority residential buildings	Method: Pre- vs posttest surveys, survey 6 months after the training Participants experienced reduced anxiety concerning computers and increased confidence in locating health information.
Xie and Bugg [84]	To teach older adults to access and use high-quality internet-based health information	Educational program: 2-hour sessions twice a week over 4 weeks. The maximum number of participants per workshop was 7. Total No. of participants: 100	Public libraries	Method: Pre- vs posttest surveys Participants showed significantly reduced computer anxiety (*P*<.001), increased interest in computers (*P*=.001), and improved efficacy (*P*<.001) from pretraining to posttraining.
Chu and Mastel-Smith [85]	To enhance older adults’ ability to grasp and manage health-related information retrieved from the internet and act accordingly	Educational program: 2-hour sessions once a week over 5 weeks. No. of participants: 12	A parish-sponsored, older adult leisure learning center	Method: Pre- vs posttest surveys; survey conducted 6 weeks after the training Participants experienced reduced anxiety, increased confidence, and a sense of self-efficacy at the end of the 5-week program and 6 weeks after program completion (*P*<.001).
Fink and Beck [86]	To improve the eHealth literacy of adults aged 50 years and older	Educational programs: 70 minutes to complete an educational online program and answer questions. No. of participants: 64	Internet-based setting	Method: Experimental group vs control group survey comparison Compared to the control group, the experimental group participants rated higher usability and learned more information on a new website.

^a^OHIS: online health information seeking.

^b^YWCA: Young Women's Christian Association.

^c^MHLC: multidimensional health locus of control.

^d^HOS: health opinion survey.

Further, 4 of the 11 programs were guided by established theories, models, or concepts (eg, the self-efficacy theory and the health belief model). All the studies involved some form of evaluation, including postsession surveys or interviews, pre- versus postintervention comparisons, and experimental versus control group comparisons. In addition, 5 studies evaluated the effectiveness of the intervention outcomes from a longitudinal perspective over a period ranging from 1 month to 1 year to the competence of the program. Among all the studies, 9 statistically assessed the effects of the intervention. Measures varied across the studies; these included opinions from surveys on the internet, self-efficacy in seeking health information, and anxiety regarding computer use. All the articles reported some positive outcomes of the intervention programs.

## Discussion

### Principal Findings

**Figure 3:**
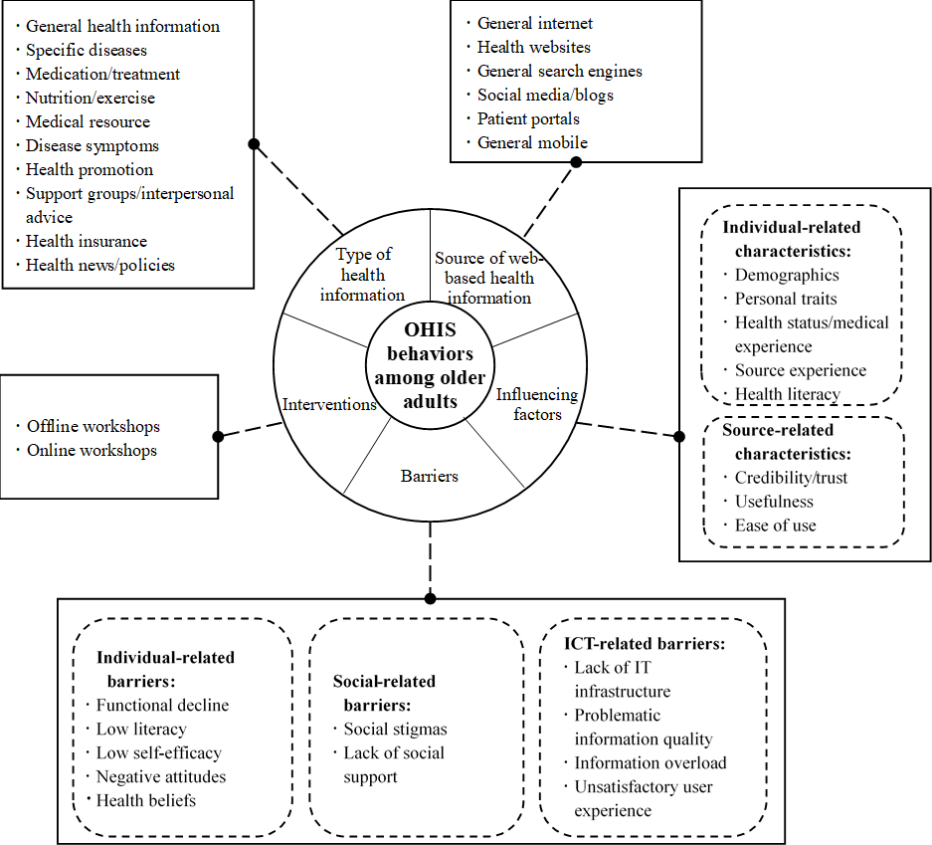
Overview of principal findings. ICT: information and communication technology; IT: information technology; OHIS: online health information seeking.

This systematic scoping review provides an overview of OHIS behaviors among older adults, as shown in Figure 3. Overall, the findings of this paper reveal core elements of OHIS among older adults. First, the types and sources of health information that older adults search for were clearly presented. Then, a portion of the studies explored the main factors influencing older adults' OHIS behaviors, which can be categorized as individual-related and source-related characteristics. Then, we identified the barriers to OHIS behavior in older adults from existing literature, including individual barriers, social barriers, and ICT barriers. Finally, this paper provides an in-depth analysis of the interventions mentioned in some of the included papers to support OHIS behaviors among older adults. We believe that the framework of this paper can, to some extent, help researchers to better position their research objectives in future studies so that the objectives correspond to specific dimensions for in-depth empirical investigation.

Regarding the first research question, the results show that older adults sought various types of health information on the internet, including information about specific diseases, medication and treatment, nutrition and exercise, medical resources, disease symptoms, health promotion, support groups and interpersonal advice, health insurance information, and health news or policies. The information sources included health websites, general search engines, social media and blogs, patient portals, and mobile devices. The types of health information sought differed from those that interest young people. According to a recent systematic review [87], adolescents and youths (<24 years) search the internet for daily health-related issues, physical and psychological well-being, sexual health, social problems, and culturally sensitive topics. Compared to the adolescent and youth population, older adults tend to search more for disease-related health information topics.

As for the second research question, the results point to 2 main types of factors influencing older adults’ OHIS: individual-related characteristics and source-related characteristics. The individual-related characteristics include demographics, anxiety, beliefs, attitudes, self-efficacy, personality, health status, medical history, health care service availability, source experience, health literacy, and motivations. Among the source-related characteristics, credibility, usefulness, and trust were the 3 factors most frequently mentioned in the studies. We noted that the primary factors influencing older adults’ OHIS differ from those influencing young adults. A systematic review of studies investigating young adults’ (<24 years) OHIS [87] revealed that the most frequently mentioned influencing factors were gender, age, educational status, emotional characteristics, engagement in risky behaviors, and eHealth literacy.

The results for the third research question reveal that older adults might encounter 3 types of barriers during their OHIS, including individual barriers (eg, low literacy), social barriers (eg, social stigmas), and ICT-related barriers (eg, lack of ICT devices). These barriers may hinder effective OHIS behaviors of older adults. The results suggest some differences from the findings on young adults’ OHIS. For the adolescent and youth population (<24 years), the main barriers to OHIS include online privacy and concerns about information credibility [87]. Although some studies report low health literacy among adolescents [88], older adults seem to have more difficulties in this respect than adolescents [89,90].

As for the fourth research question, the review found that many intervention programs have been created to support older adults’ OHIS; they primarily use educational training workshops in offline and online formats. Most training programs contained multiple sessions, with each session lasting 2 to 3 hours; the duration of the programs varied from 1 to 4 months, and all the programs reported at least some positive effects in support of older adults’ OHIS.

### Implications for Future Research

Overall, this systematic scoping review identified the need for more in-depth research on older adults’ OHIS. As can be seen from the aforementioned evidence, a subset of studies have treated OHIS as a variable or construct and focused on exploring the factors influencing OHIS in older adults. Other studies treat OHIS as a process and investigate how the older adults search the internet for health information. However, given the complexity of the health conditions of older people and a projected future intensification of information overload, older adults will encounter more serious problems when searching for health information on the internet, such as how to select from among multimodal information sources, how to express health information needs, and how to evaluate health misinformation. Considering the growing population of older adults, the importance of internet-based information seeking for overall public health, and the lack of best practices, more research on this topic is needed. In this section, we propose several directions for future research based on gaps identified in the review.

#### Investigations on the OHIS Behavior of Older Adults Above 85 Years

With the accelerating pace of global aging, the population of older adults is steadily growing. Instead of classifying the large population of older adults as one group, researchers are advocating for a more precise segmentation of this population, such as the youngest-old (65 to 74 years), middle-old (75 to 84 years), and oldest-old groups (above 85 years) [91]. Regarding OHIS, the age distribution of the samples in this systematic scoping review indicates that the exploration of OHIS by the oldest-old group is very limited [92]. Most articles included in this review have focused on the youngest- and middle-old groups [30], whereas there is a lack of research on the health information needs and behaviors of the oldest-old group. Future OHIS research can be appropriately skewed toward the oldest-old group to consider the physiological and psychological characteristics, the unique information needs, and explore the influences, processes, and health outcomes of the OHIS of this group more empirically within the framework of everyday information mastering [93].

#### Conducting More Longitudinal, Action, and Mixed Methods Research

As for research methods, most current studies use cross-sectional data collection methods and pay little attention to longitudinal approaches. In future, more consideration can be given to the adoption of longitudinal methods, such as the experience sampling method and the ethnographic approach. In particular, for intervention studies on OHIS behaviors in older adults, educational training programs with long time spans could provide data to improve OHIS performance and the health literacy of older adults. More participatory action research at the community level would enrich the network of actors in OHIS for older adults and engage more participants, thereby promoting interdisciplinary and collaborative health information practices in this population. In addition, future studies might consider more mixed methods approaches to leverage the advantages of qualitative and quantitative approaches and triangulate primary data with secondary data. Existing mixed methods studies have been conducted mainly based on quantitative questionnaire analyses as well as qualitative focus groups, and a richer mix of methods is to be further explored for this topic in future. Finally, as prior studies have relied heavily on self-reported data, future studies could consider more behavioral data using methods such as eye-tracking and electroencephalograms.

#### Elaboration on Mobile Context and Cross-platform Scenario of Older Adults’ OHIS

Information types and information sources are the essential contextual factors in OHIS [94-96]. However, this review found that most studies on older adults’ OHIS do not clearly explain what health-related information was involved or from where the information was gathered. In terms of information types, current studies mainly focus on searches for disease and treatment information. More studies are needed to address other types of health information that older adults might seek, such as information on environmental health and disease prevention.

Regarding information sources, studies are needed to investigate older adults’ use of mobile devices for OHIS. With the development of the mobile internet and the internet of things, OHIS scenarios for older adults are changing. Mobile device–based health information access can more effectively meet the health information needs of older adults, facilitate daily health monitoring and self-tracking, and improve context-driven, health-related decision-making among older adults. For example, increasing numbers of older adults are seeking health information on their smart phones through short video apps like TikTok [97,98]. Furthermore, in addition to searching for health information on their mobile devices, increasing numbers of older adults are using mobile social apps to create content [99]. Future research could focus more on the relationship between OHIS and health-related content generation by older adults.

In addition, further exploration of complicated OHIS scenarios is needed. For example, with the popularity of wearable devices and the development of various health-related vertical search platforms, a portion of the older adult population with higher information literacy will become more proficient at searching for a full range of health information using various smart devices and immersive technologies [100], such as interacting with information through voice recognition and gesture control. Thus, explorations of cross-platform and cross-device seeking behaviors in OHIS by older adults are needed. Meanwhile, in addition to active information seeking, more types of seeking behaviors, such as passive exposure, information encountering, and surrogate health information seeking [101,102], deserve attention and further investigation. In particular, the influences and positive outcomes of searching as learning during OHIS by older adults is a topic worth exploring.

#### Facilitating Older Adults’ OHIS by Explicating Technology Affordance

This review revealed that current research on factors influencing OHIS in older adults focuses more on demographic issues and individual-related characteristics than on source-related factors. In recent years, increased emphasis has been placed on aging-friendly designs in human-computer interaction [103], and the user experience–oriented design of various social apps and smart devices is centered on the needs and behavioral preferences of older adults, with an interest in meeting their personalized requirements. We believe that the affordance of technology in aging-friendly design is also a highly influential factor for promoting OHIS in older adults. It would be fruitful to integrate the uses and gratifications theory with the affordance lens to better promote the positive impact of new media platforms on older adults’ information-seeking behaviors [104,105]. More attention needs to be placed on the ease of use, usability, and sociability of aging-friendly information sources and information systems. In particular, in the upcoming human-centered artificial intelligence era, older people’s perception of the trustworthiness of multimodal information sources and their trust in algorithm-based content recommendations will continue to change. Therefore, the age-appropriate design of OHIS needs to constantly break away from stereotypes of older people and re-establish a more adaptive mental model. The lens of the affordance theory could be applied to help situate OHIS for older adults in the context of information practices, promoting deep reflection on the interaction of actors with sociocultural environments and on the mediated nature of technology [106]. For instance, an OHIS platform should provide rich technology affordances for older adults and provide targeted support for active health information access, information encounters, and information avoidance problems in different sociocultural environments. Future research could focus more on how technology affordance can better mediate older adults' OHIS gratification by attempting to build a more detailed affordance typology [107]—such as handling, effecter, and motivational affordances—to measure older adults' gratifications for OHIS using social media.

#### Promoting and Measuring the Performance of OHIS Interventions for Older Adults

The results show that older adults encounter many barriers in OHIS; thus, many intervention programs have been created to support their searching. However, current intervention programs still leave considerable room for improvement. First, current educational training programs are generally small-scale ones, making it difficult to reach a wide group of older adults; most programs are offline workshops, and there are few internet-based programs. Future OHIS interventions for older adults need to offer more technology-mediated web-based programs and provide richer formats than workshops and tutorials, such as distance education for older adults using gamification and immersive technology. Moreover, most current intervention programs operate in the United States; older adults living in less developed countries or areas received less attention. Future studies on OHIS in older adults must involve more trans- and cross-national, or regional and cross-cultural comparative studies to further explore the influence of sociocultural factors on older adults' OHIS behaviors. We also recommend that more information and communication technology for development (known as “ICT4D”) projects focus on upgrading OHIS and improving the same for older adults [108], thereby better promoting health literacy and health mobility for older adults in developing countries and regions.

In particular, researchers need to draw more on the design science research paradigm. Design science research is an innovative and often iterative problem-solving process that builds and evaluates artifacts [109]. In our research context, the purposeful artifacts could be search systems, training courses, workshops, tutorials, or citizen science programs. In the building phase of artifact development, most units of analysis relate to offline workshops and neglect other types of artifacts. It is also noteworthy that current intervention studies lack a theoretical lens, and only a few studies have designed interventions based on theoretical foundations. Future interventions for older adults’ OHIS need to embrace the theoretical considerations that design science research has been advocating [110]. In the evaluation phase of artifact development, current studies lack long-term assessments of intervention effects. Future studies should consider more participatory action research to iteratively test the effects of OHIS interventions on older adults and select some specific health domains—such as chronic diseases, cancer, and mental health—for attempting to verify the actual effects of OHIS interventions on information literacy, health literacy, and health outcomes of older adults. In addition, future studies could contemplate providing various forms of support based on the perspectives of older users, allowing them to participate in the project design process and thus help them overcome search barriers.

### Limitations

This systematic scoping review has several limitations. The first one is in terms of search sources. Owing to the interdisciplinary nature of OHIS research in older adults, although we tried to search multiple databases using relevant keywords and consulted academic librarians to improve our search strategy, it was nevertheless inevitable that some literature would be missed, especially relevant research in unofficially published conference proceedings. The backward and forward strategy can be further used to expand the literature search sources in future [111]. Second, in terms of the literature type, this review mainly focuses on empirical studies, whereas some opinion papers, descriptive cases, and short communications on OHIS for older adults were excluded from our literature pool, and some complementary analyses of such nonresearch articles can be conducted in future. Finally, in terms of the analytical approach for searching literature, this study did not conduct a comparative chronological analysis of the literature in different periods, which to a certain extent could not fully reveal the impact of technological and sociocultural changes on older adults’ OHIS behavior. In future, the introduction of knowledge graphs can be considered to map the themes of the literature at different stages.

### Conclusions

This review provides an overview of how older adults’ OHIS has been studied. It reveals that older adults search for various types of health information on the internet using different types of web-based sources and that their OHIS is jointly influenced by source-related and individual-related factors. Their difficulties in searching arise from individual, social, and ICT-related barriers. Some educational intervention programs that support older adults’ OHIS have been initiated in the form of web-based and offline workshops. Furthermore, the review reveals that the topic of older adults’ OHIS is understudied, although the number of studies is increasing. Nevertheless, more studies are needed to understand the problems associated with older adults’ interactions with health information and better support them in their decision-making when they are searching for medical and health information on the internet. Based on the findings of the review, the authors propose several objectives for future research.

## Appendix

Multimedia Appendix 1 Database search strategies.

Multimedia Appendix 2 Overview of included studies.

## Abbreviations

ICT: information and communication technology
OHIS: online health information seeking
