# Supplementary material for: Online Health Information Seeking Behaviors Among Older Adults: Systematic Scoping Review
Source: J Med Internet Res. 2022 Feb 16;24(2):e34790. doi: 10.2196/34790 (PMC8892316; doi:10.2196/34790)
Supplement: Multimedia Appendix 1 [file jmir_v24i2e34790_app1.docx]

Appendix 1. Database search strategies

| **Database** | **Search fields** | **Search strategy** |
| --- | --- | --- |
| PubMed | Title/ Abstract | (“older adults” OR elderly OR aging OR senior OR seniors OR “older people” OR “aged 60” OR “aged 65”) AND (find* OR search* OR seek* OR access* OR retriev*) AND (internet OR online OR web) AND “information” AND (“health” OR “medicine” OR “drug” OR “wellness” OR “illness”) |
| Cochrane Library | Title/ Abstract/ Keywords | (“older adults” OR elderly OR aging OR senior OR seniors OR “older people” OR “aged 60” OR “aged 65”) AND (find* OR search* OR seek* OR access* OR retriev*) AND (internet OR online OR web) AND “information” AND (“health” OR “medicine” OR “drug” OR “wellness” OR “illness”) |
| APA PsycINFO  CINAHL Plus with Full Text  Library &Information Science Source  Medline  Psychology and Behavioral Sciences Collection  ABI/INFORM  Communication & Mass Media Complete  Library, Information Science & Technology Abstracts  ACM Digital Library | Abstract | (“older adults” OR elderly OR aging OR senior OR seniors OR “older people” OR “aged 60” OR “aged 65”) AND (find* OR search* OR seek* OR access* OR retriev*) AND (internet OR online OR web) AND “information” AND (“health” OR “medicine” OR “drug” OR “wellness” OR “illness”) |
| Web of Science | Topic | (“older adults” OR elderly OR aging OR senior OR seniors OR “older people” OR “aged 60” OR “aged 65”) AND (find* OR search* OR seek* OR access* OR retriev*) AND (internet OR online OR web) AND “information” AND (“health” OR “medicine” OR “drug” OR “wellness” OR “illness”) |
