# Supplementary material for: Online Health Information Seeking Behaviors Among Older Adults: Systematic Scoping Review
Source: J Med Internet Res. 2022 Feb 16;24(2):e34790. doi: 10.2196/34790 (PMC8892316; doi:10.2196/34790)
Supplement: Multimedia Appendix 2 [file jmir_v24i2e34790_app2.docx]

**Appendix 2. Overview of included studies**

| **Authors (year)** | **Journal/ Conference** | **Country** | **Sample size** | **Age range** | **Methods** |
| --- | --- | --- | --- | --- | --- |
| Gollop (1997) [1] | Bulletin of the Medical Library Association | U.S. | 50 | 63-88 | Qualitative |
| Alpay et al. (2004) [2] | Health Informatics Journal | Netherlands | 18 | 60+ | Qualitative |
| Campbell (2005) [3] | Perspectives in health information management/AHIMA | U.S. | 70 | 60-83 | Quantitative |
| Campbell & Nolfi (2005) [4] | Journal of medical Internet research | U.S. | 42 | -- | Quantitative |
| Meischke et al. (2005) [5] | Heart & Lung | U.S. | 316 | 65+ | Quantitative |
| Tak and Hong (2005) [6] | Orthopaedic Nursing | U.S. | 71 | 60+ | Quantitative |
| Flynn et al. (2006) [7] | Journal of general internal medicine | U.S. | 4528 | 63-66 | Quantitative |
| Hoffman-Goetz et al. (2006) [8] | Journal of Consumer Health on the Internet | Canada | 44 | 50+ | Quantitative |
| Bertera et al. (2007) [9] | Educational Gerontology | U.S. | 42 | 55-71 | Mixed methods |
| Given et al. (2007) [10] | Journal of the U.S.n Society for Information Science and Technology | Canada | 12 | 65 | Qualitative |
| Hardt & Hollis-Sawyer (2007) [11] | Educational Gerontology | U.S. | 43 | 55+ | Mixed methods |
| Leung et al. (2007) [12] | Public Health Nursing | China | 88 | 50+ | Quantitative |
| McMillan & Macias (2008) [13] | Journal of health communication | U.S. | 424 | 55+ | Quantitative |
| Campbell (2009) [14] | Home Health Care Management & Practice | U.S. | 36 | -- | Quantitative |
| Chu et al. (2009) [15] | Journal of the Medical Library Association: JMLA | U.S. | 112 | 65+ | Quantitative |
| Czaja et al. (2009) [16] | Proceedings of the Human Factors and Ergonomics Society Annual Meeting | U.S. | 112 | 50-85 | Quantitative |
| Taha et al. (2009) [17] | The Gerontologist | U.S. | 53 | 51-85 | Mixed methods |
| Xie (2009) [18] | Journal of health communication | U.S. | 20 | 60+ | Qualitative |
| Xie & Bugg (2009) [19] | Library & information science research | U.S. | 131 | 54-89 | Quantitative |
| Chu & Mastel-Smith (2010) [20] | CIN: Computers, Informatics, Nursing | U.S. | 12 | 65+ | Qualitative |
| Czaja et al. (2010) [21] | Gerontechnology | U.S. | 40 | 60-83 | Quantitative |
| Nguyen et al. (2010) [22] | Journal of health communication | U.S. | 104 | 50-70 | Qualitative |
| Harrod (2010) [23] | Ageing International | U.S. | 8 | 58-72 | Qualitative |
| Chung et al. (2011) [24] | International journal of older people nursing | U.S. | 91 | 65+ | Mixed methods |
| Mayoh et al. (2011) [25] | Indo-Pacific Journal of Phenomenology | U.K. | 6 | 60+ | Qualitative |
| Robertson-Lang et al. (2011) [26] | Canadian Journal on aging/La Revue canadienne du vieillissement | Canada | 83 | 55-86 | Quantitative |
| Zulman et al. (2011) [27] | Journal of medical Internet research | U.S. | 823 | 50+ | Quantitative |
| Crabb et al. (2012) [28] | Gerontology | U.S. | 50 | 65+ | Quantitative |
| Cresci et al. (2012) [29] | Educational Gerontology | U.S. | 149 | 60+ | Quantitative |
| Huang et al. (2012) [30] | Proceedings of the 2012 iConference | U.S. | 17 | 56-78 | Mixed methods |
| Lam and Lam (2012) [31] | Electronic journal of health informatics | Australia | 1815 | 60-74 | Quantitative: |
| Zajac et al. (2012) [32] | The Australasian medical journal | Australia | 8762 | 50-74 | Quantitative |
| Chaudhuri et al. (2013) [33] | Computers, informatics, nursing: CIN | U.S. | 403 | 57-100 | Quantitative |
| Li et al. (2013) [34] | Journal of medical Internet research | U.S. | 195 | -- | Quantitative |
| Sheng and Simpson (2013) [35] | Cyberpsychology, Behavior, and Social Networking | U.S. | 771 | 50+ | Quantitative |
| Chang and Im (2014) [36] | Geriatric Nursing | Korea | 300 | 55+ | Quantitative |
| Chang et al. (2014) [37] | Journal of women & aging | Singapore | 22 | 61-79 | Qualitative |
| Goodall et al. (2014) [38] | European journal of cancer care | Australia | 54 | 63-94 | Qualitative |
| Wong et al. (2014) [39] | Journal of Applied Gerontology | China | 98 | 55-91 | Quantitative |
| Fink & Beck (2015) [40] | Journal of Applied Gerontology | U.S. | 64 | 50+ | Mixed methods |
| Sanders et al. (2015) [41] | Public Relations Review | Spain | 117 | 55-81 | Qualitative |
| Sheng and Simpson (2015) [42] | Health Marketing Quarterly | U.S. | 832 | -- | Quantitative |
| Tennant et al. (2015) [43] | Journal of medical Internet research | U.S. | 283 | 50+ | Quantitative |
| Cajita et al. (2016) [44] | Gerontechnology | U.S. | 1945 | 65+ | Quantitative |
| Parida et al. (2016) [45] | Psychology & Marketing | Sweden | 610 | 60+ | Quantitative |
| Wu & Li (2016) [46] | Library & Information Science Research | China | 20 | 55+ | Mixed methods |
| Hong and Cho (2017) [47] | Journals of Gerontology Series B: Psychological Sciences and Social Sciences | U.S. | 6603 | 55+ | Quantitative |
| Jensen et al. (2017) [48] | Health informatics journal | U.S. | 209 | 50-75 | Quantitative |
| Leung et al. (2017) [49] | Geriatrics | China | 224 | 60+ | Quantitative |
| Lumpkins et al. (2017) [50] | Health communication | U.S. | 2108 | 50+ | Quantitative |
| Malone et al. (2017) [51] | Journal of Consumer Health on the Internet | U.S. | 110 | -- | Mixed methods |
| Sanchiz et al. (2017) [52] | Information Processing & Management | France | 20 | 60-77 | Quantitative |
| Walker et al. (2017) [53] | The Gerontologist | U.S. | 30 | 75+ | Qualitative |
| Burns et al. (2018) [54] | Health Promotion Journal of Australia | Australia | 4060 | 55+ | Quantitative |
| Cutilli et al. (2018) [55] | Orthopaedic Nursing | U.S. | 2668 | 65+ | Quantitative |
| Huvila et al. (2018) [56] | Information processing & management | Sweden | 161 | 51+ | Quantitative |
| Kim & Utz (2018) [57] | Nursing & health sciences | Korea | 129 | 65+ | Quantitative |
| Lyons et al. (2018) [58] | Educational Gerontology | Australia | 686 | 60+ | Quantitative |
| Marinescu & Rodat (2018) [59] | Romanian Journal of Communication and Public Relations | Romania | 20 | 65+ | Qualitative |
| Moult et al. (2018) [60] | BMC family practice | U.K. | 18 | 65-71 | Qualitative |
| Oh et al. (2018) [61] | Social work in public health | Korea | 235 | 60+ | Quantitative |
| Pálsdóttir (2018) [62] | Romanian Journal of Library and Information Science | Iceland | 176 | 60+ | Quantitative |
| Sak & Schulz (2018) [63] | JMIR cardio | Switzerland | 101 | 60+ | Quantitative |
| Turner et al. (2018) [64] | AMIA annual symposium proceedings | U.S. | 88 | 60+ | Qualitative |
| Zapata et al. (2018) [65] | Clinical gerontologist | U.S. | 20 | 60+ | Qualitative |
| Choi (2019) [66] | Library & Information Science Research | U.S. | 21 | 61-80 | Qualitative |
| Jin et al. (2019) [67] | Patient education and counseling | U.S. | 240 | 55-75 | Quantitative |
| Marcu et al. (2019) [68] | Journal of medical Internet research | U.K. | 56 | 50+ | Qualitative |
| Ortiz-Dowling et al. (2019) [69] | Psychology of Men & Masculinities | U.S. | 14 | 65-80 | Qualitative |
| Agyemang-Duah et al. (2020) [70] | BMC Public Health | Ghana | 30 | 65+ | Qualitative |
| Choi et al. (2020) [71] | Journal of Medical Internet Research | U.S. | 2866 | 65+ | Quantitative |
| Huisman et al. (2020a) [72] | Information, Communication & Society | Belgium | 40 | 50-80 | Qualitative |
| Huisman et al. (2020b) [73] | Journal of aging and health | Belgium | 40 | 50-80 | Qualitative |
| Sedrak et al. (2020) [74] | Journal of medical Internet research | U.S. | 72806 | 65+ | Quantitative |
| Weber et al. (2020) [75] | Journal of medical Internet research | Germany | 701 | 60+ | Quantitative |

**References:**

1. Gollop CJ. Health information seeking behavior and older african american women. Bulletin of the Medical Library Association. 1997;85(2):141-6.

2. Alpay LL, Toussaint PJ, Ezendam NP, Rövekamp TA, Graafmans WC, Westendorp RG. Easing Internet access of health information for elderly users. Health Informatics Journal. 2004;10(3):185-94. doi: 10.1177/1460458204045416.

3. Campbell RJ. Consumer informatics: elderly persons and the internet. Perspectives in health information management/AHIMA, American Health Information Management Association. 2005;2.

4. Campbell RJ, Nolfi DA. Teaching elderly adults to use the Internet to access health care information: before-after study. Journal of medical Internet research. 2005;7(2):e19.

5. Meischke H, Eisenberg M, Rowe S, Cagle A. Do older adults use the Internet for information on heart attacks? Results from a survey of seniors in King County, Washington. Heart & Lung. 2005;34(1):3-12.

6. Tak SH, Hong SH. Use of the Internet for health information by older adults with arthritis. Orthopaedic Nursing. 2005;24(2):134-8.

7. Flynn KE, Smith MA, Freese J. When do older adults turn to the internet for health information? Findings from the Wisconsin Longitudinal Study. Journal of general internal medicine. 2006;21(12):1295-301.

8. Hoffman-Goetz L, Friedman DB, Celestine A. Evaluation of a public library workshop: Teaching older adults how to search the Internet for reliable cancer information. Journal of Consumer Health On the Internet. 2006;10(3):29-43. doi: 10.1300/J381v10n03_03.

9. Bertera EM, Bertera RL, Morgan R, Wuertz E, Attey AMO. Training Older Adults to Access Health Information. Educational Gerontology. 2007;33(6):483-500. doi: 10.1080/03601270701328250.

10. Given LM, Ruecker S, Simpson H, Sadler E, Ruskin A. Inclusive interface design for seniors: Image-browsing for a health information context. Journal of the American Society for Information Science and Technology. 2007;58(11):1610-7. doi: 10.1002/asi.20645.

11. Hardt JH, Hollis-Sawyer L. Older Adults Seeking Healthcare Information on the Internet. Educational Gerontology. 2007;33(7):561-72. doi: 10.1080/03601270701364628.

12. Leung A, Ko P, Chan KS, Chi I, Chow N. Searching health information via the web: Hong Kong Chinese older adults' experience. Public Health Nursing. 2007;24(2):169-75.

13. McMillan SJ, Macias W. Strengthening the safety net for online seniors: factors influencing differences in health information seeking among older internet users. Journal of health communication. 2008 Dec;13(8):778-92. PMID: 19051113. doi: 10.1080/10810730802487448.

14. Campbell RJ. Internet-based health information seeking among low-income, minority seniors living in urban residential centers. Home Health Care Management & Practice. 2009;21(3):195-202.

15. Chu A, Huber J, Mastel-Smith B, Cesario S. "Partnering with Seniors for Better Health": computer use and Internet health information retrieval among older adults in a low socioeconomic community. J Med Libr Assoc. 2009 Jan;97(1):12-20. PMID: 19159002. doi: 10.3163/1536-5050.97.1.003.

16. Czaja SJ, Sharit J, Nair SN, Lee CC, editors. Older adults and internet health information seeking. Proceedings of the Human Factors and Ergonomics Society Annual Meeting; 2009.

17. Taha J, Sharit J, Czaja S. Use of and Satisfaction With Sources of Health Information Among Older Internet Users and Nonusers. The Gerontologist. 2009;49(5):663-73. doi: 10.1093/geront/gnp058.

18. Xie B. Older adults' health information wants in the internet age: Implications for patient–provider relationships. Journal of health communication. 2009 Sep;14(6):510-24. PMID: 19731124. doi: 10.1080/10810730903089614.

19. Xie B, Bugg JM. Public library computer training for older adults to access high-quality Internet health information. Library & information science research. 2009 Sep 1;31(3):155-62. PMID: 20161649. doi: 10.1016/j.lisr.2009.03.004.

20. Chu A, Mastel-Smith B. The outcomes of anxiety, confidence, and self-efficacy with Internet health information retrieval in older adults: a pilot study. CIN: Computers, Informatics, Nursing. 2010;28(4):222-8.

21. Czaja SJ, Sharit J, Hernandez MA, Nair SN, Loewenstein D. Variability among older adults in Internet health information-seeking performance. Gerontechnology. 2010;9(1):46-55. doi: 10.4017/gt.2010.09.01.004.00.

22. Nguyen GT, Shungu NP, Niederdeppe J, Barg FK, Holmes JH, Armstrong K, et al. Cancer-related information seeking and scanning behavior of older Vietnamese immigrants. Journal of health communication. 2010;15(7):754-68.

23. Harrod M. “I Have to Keep Going”: Why Some Older Adults Are Using the Internet for Health Information. Ageing International. 2010;36(2):283-94. doi: 10.1007/s12126-010-9090-z.

24. Chung J, Gassert CA, Kim HS. Online health information use by participants in selected senior centres in Korea: current status of internet access and health information use by Korean older adults. International Journal of Older People Nursing. 2011;6(4):261-71. doi: 10.1111/j.1748-3743.2010.00238.x.

25. Mayoh J, Todres L, Bond CS. Exploring the Online Health Information Seeking Experiences of Older Adults. Indo-Pacific Journal of Phenomenology. 2011;11(2):1-13. doi: 10.2989/ipjp.2011.11.2.3.1162.

26. Robertson-Lang L, Major S, Hemming H. An exploration of search patterns and credibility issues among older adults seeking online health information. Canadian Journal on aging/La Revue canadienne du vieillissement. 2011 Dec;30(4):631-45. PMID: 22085455. doi: 10.1017/S071498081100050X.

27. Zulman DM, Kirch M, Zheng K, An LC. Trust in the internet as a health resource among older adults: analysis of data from a nationally representative survey. Journal of medical Internet research. 2011;13(1):e19.

28. Crabb RM, Rafie S, Weingardt KR. Health-Related Internet Use in Older Primary Care Patients. Gerontology. 2012;58(2):164-70. doi: 10.1159/000329340.

29. Cresci MK, Jarosz PA, Templin TN. Are health answers online for older adults? Educational Gerontology. 2012;38(1):10-9.

30. Huang M, Hansen D, Xie B, editors. Older adults' online health information seeking behavior. Proceedings of the 2012 iConference; 2012.

31. Lam MK, Lam LT. Health information-seeking behaviour on the Internet and health literacy among older Australians. Electronic journal of health informatics. 2012.

32. Zajac IT, Flight IH, Wilson C, Turnbull D, Cole S, Young G. Internet usage and openness to internet-delivered health information among Australian adults aged over 50 years. The Australasian medical journal. 2012;5(5):262.

33. Chaudhuri S, Le T, White C, Thompson H, Demiris G. Examining health information-seeking behaviors of older adults. Comput Inform Nurs. 2013;31(11):547-53. PMID: 23974574.

34. Li Y, Polk J, Plankey M. Online health-searching behavior among HIV-seropositive and HIV-seronegative men who have sex with men in the Baltimore and Washington, DC area. Journal of medical Internet research. 2013;15(5):e78.

35. Sheng X, Simpson PM. Seniors, health information, and the Internet: motivation, ability, and Internet knowledge. Cyberpsychology, Behavior, and Social Networking. 2013;16(10):740-6.

36. Chang SJ, Im E-O. A path analysis of Internet health information seeking behaviors among older adults. Geriatric Nursing. 2014;35(2):137-41.

37. Chang L, Basnyat I, Teo D. Seeking and processing information for health decisions among elderly Chinese Singaporean women. Journal of women & aging. 2014;26(3):257-79. PMID: 24919105. doi: 10.1080/08952841.2014.888881.

38. Goodall KT, Newman LA, Ward PR. Improving access to health information for older migrants by using grounded theory and social network analysis to understand their information behaviour and digital technology use. European Journal of Cancer Care. 2014;23(6):728-38. doi: 10.1111/ecc.12241.

39. Wong CK, Yeung DY, Ho HC, Tse K-P, Lam C-Y. Chinese older adults’ Internet use for health information. Journal of Applied Gerontology. 2014;33(3):316-35.

40. Fink A, Beck JC. Developing and Evaluating a Website to Guide Older Adults in Their Health Information Searches: A Mixed-Methods Approach. J Appl Gerontol. 2015 Aug;34(5):633-51. PMID: 24652883. doi: 10.1177/0733464813486961.

41. Sanders K, Sánchez Valle M, Viñaras M, Llorente C. Do we trust and are we empowered by “Dr. Google”? Older Spaniards’ uses and views of digital healthcare communication. Public Relations Review. 2015;41(5):794-800. doi: 10.1016/j.pubrev.2015.06.015.

42. Sheng X, Simpson PM. Health care information seeking and seniors: determinants of Internet use. Health Marketing Quarterly. 2015;32(1):96-112.

43. Tennant B, Stellefson M, Dodd V, Chaney B, Chaney D, Paige S, et al. eHealth literacy and Web 2.0 health information seeking behaviors among baby boomers and older adults. Journal of medical Internet research. 2015;17(3):e70.

44. Cajita MI, Whitehouse E, Budhathoki C, Hodgson N. Association between Internet use and decision-making preference in older adults. Gerontechnology. 2016;14(2):97-104. PMID: 28190988. doi: 10.4017/gt.2016.14.2.008.00.

45. Parida V, Mostaghel R, Oghazi P. Factors for elderly use of social media for health‐related activities. Psychology & Marketing. 2016;33(12):1134-41.

46. Wu D, Li Y. Online health information seeking behaviors among Chinese elderly. Library & Information Science Research. 2016;38(3):272-9. doi: 10.1016/j.lisr.2016.08.011.

47. Hong YA, Cho J. Has the digital health divide widened? Trends of health-related internet use among older adults from 2003 to 2011. Journals of Gerontology Series B: Psychological Sciences and Social Sciences. 2017;72(5):856-63.

48. Jensen JD, Liu M, Carcioppolo N, John KK, Krakow M, Sun Y. Health information seeking and scanning among US adults aged 50–75 years: Testing a key postulate of the information overload model. Health informatics journal. 2017;23(2):96-108.

49. Leung DY, Chow TT, Wong EM. Cancer-related information seeking and scanning behaviors among older Chinese adults: examining the roles of fatalistic beliefs and fear. Geriatrics. 2017;2(4):38.

50. Lumpkins CY, Mabachi N, Lee J, Pacheco C, Greiner KA, Geana M. A prescription for internet access: appealing to middle-aged and older racial and ethnic minorities through social network sites to combat colorectal cancer. Health communication. 2017;32(7):916-20.

51. Malone T, Jo P, Clifton S. Perceived eHealth Literacy and Information Behavior of Older Adults Enrolled in a Health Information Outreach Program. Journal of Consumer Health on the Internet. 2017;21(2):137-47. doi: 10.1080/15398285.2017.1300040.

52. Sanchiz M, Chin J, Chevalier A, Fu WT, Amadieu F, He J. Searching for information on the web: Impact of cognitive aging, prior domain knowledge and complexity of the search problems. Information Processing & Management. 2017 2017/01/01/;53(1):281-94. doi: <https://doi.org/10.1016/j.ipm.2016.09.003>.

53. Walker J, Crotty BH, O’Brien J, Dierks MM, Lipsitz L, Safran C. Addressing the Challenges of Aging: How Elders and Their Care Partners Seek Information. The Gerontologist. 2017;57(5):955-62. doi: 10.1093/geront/gnw060.

54. Burns P, Jones SC, Caputi P, Iverson D. Are older Australians with chronic diseases online? Health Promotion Journal of Australia. 2018;29(1):72-8.

55. Cutilli CC, Simko LC, Colbert AM, Bennett IM. Health Literacy, Health Disparities, and Sources of Health Information in US Older Adults. Orthopaedic Nursing. 2018;37. doi: 10.1097/NOR.0000000000000418.

56. Huvila I, Enwald H, Eriksson-Backa K, Hirvonen N, Nguyen H, Scandurra I. Anticipating ageing: Older adults reading their medical records. Information Processing & Management. 2018;54(3):394-407. doi: 10.1016/j.ipm.2018.01.007.

57. Kim SH, Utz S. Association of health literacy with health information‐seeking preference in older people: A correlational, descriptive study. Nursing & health sciences. 2018;20(3):355-60.

58. Lyons A, Mikolajczak G, Heywood W, Fileborn B, Minichiello V, Hinchliff S, et al. Sources of information-seeking on sexually transmitted infections and safer sex by older heterosexual Australian men and women. Educational Gerontology. 2018;44(2-3):186-95. doi: 10.1080/03601277.2018.1433989.

59. Marinescu V, Rodat S. Romanian and German Seniors in Quest of Online Health-Related Information: An Exploratory Comparative Study. Romanian Journal of Communication and Public Relations. 2018;20(1):25-45.

60. Moult A, Burroughs H, Kingstone T, Chew-Graham CA. How older adults self-manage distress - does the internet have a role? A qualitative study. BMC family practice. 2018 Nov 29;19(1):185. PMID: 30497414. doi: 10.1186/s12875-018-0874-7.

61. Oh YS, Choi EY, Kim YS. Predictors of smartphone uses for health information seeking in the Korean elderly. Social work in public health. 2018;33(1):43-54.

62. Pálsdóttir Á. Analysing Digital Information Seeking and Quality Evaluation of Sources about Healthy Lifestyle among Senior Citizens in Iceland. Revista Română de Biblioteconomie și Știința Informării = Romanian Journal of Library and Information Science. 2018;14(2):35-45. doi: 10.26660/rrbsi.2018.14.2.35.

63. Sak G, Schulz PJ. Exploring Health Information-Seeking Preferences of Older Adults With Hypertension: Quasi-Experimental Design. JMIR Cardio. 2018 May 30;2(1):e12. PMID: 31758784. doi: 10.2196/cardio.8903.

64. Turner AM, Osterhage KP, Taylor JO, Hartzler AL, Demiris G. A closer look at health information seeking by older adults and involved family and friends: design considerations for health information technologies. Paper presented at the AMIA annual symposium proceedings. 2018;2018:1036.

65. Zapata AML, Beaudreau SA, O’Hara R, Bereknyei Merrell S, Bruce J, Garrison-Diehn C, et al. Information-Seeking about Anxiety and Perceptions about Technology to Teach Coping Skills in Older Veterans. Clinical Gerontologist. 2018;41(4):346-56. doi: 10.1080/07317115.2017.1359716.

66. Choi W. Older adults' health information behavior in everyday life settings. Library & Information Science Research. 2019 Oct;41(45). PMID: WOS:000500079400003. doi: 10.1016/j.lisr.2019.100983.

67. Jin SW, Lee Y, Dia DA. Analyzing paths from online health information seeking to colorectal cancer screening using health literacy skills frame and cognitive mediation model. Patient Education and Counseling. 2019;102(3):416-23. doi: 10.1016/j.pec.2018.11.002.

68. Marcu A, Muller C, Ream E, Whitaker KL. Online Information-Seeking About Potential Breast Cancer Symptoms: Capturing Online Behavior With an Internet Browsing Tracking Tool. J Med Internet Res. 2019 Feb 6;21(2):e12400. PMID: 30724741. doi: 10.2196/12400.

69. Ortiz-Dowling EM, Ananian CD, Larkey LK, Hooker SP. Health-Seeking Behaviors and Health Information Gathering in Older Mexican American Males. Psychology of Men & Masculinity. 2019 Oct;20(4):564-74. PMID: WOS:000484118900010. doi: 10.1037/men0000194.

70. Agyemang-Duah W, Arthur-Holmes F, Peprah C, Adei D, Peprah P. Dynamics of health information-seeking behaviour among older adults with very low incomes in Ghana: a qualitative study. BMC Public Health. 2020 2020/06/15;20(1):928. doi: 10.1186/s12889-020-08982-1.

71. Choi NG, DiNitto DM, Lee OE, Choi BY. Internet and Health Information Technology Use and Psychological Distress Among Older Adults With Self-Reported Vision Impairment: Case-Control Study. Journal of Medical Internet Research. 2020;22(6):e17294.

72. Huisman M, Joye S, Biltereyst D. Health on Wikipedia: a qualitative study of the attitudes, perceptions, and use of Wikipedia as a source of health information by middle-aged and older adults. Information, Communication & Society. 2021;24(12):1797-813.

73. Huisman M, Joye S, Biltereyst D. Searching for Health: Doctor Google and the Shifting Dynamics of the Middle-Aged and Older Adult Patient-Physician Relationship and Interaction. Journal of Aging and Health. 2020;32(8):998-1007. PMID: WOS:000491044700001. doi: 10.1177/0898264319873809.

74. Sedrak MS, Soto-Perez-De-Celis E, Nelson RA, Liu J, Waring ME, Lane DS, et al. Online health information–seeking among older women with chronic illness: analysis of the women’s health initiative. Journal of medical Internet research. 2020;22(4):e15906.

75. Weber W, Reinhardt A, Rossmann C. Lifestyle Segmentation to Explain the Online Health Information–Seeking Behavior of Older Adults: Representative Telephone Survey. Journal of medical Internet research. 2020;22(6):e15099.
